# Joint Effects of Cigarette Smoking and Green Tea Consumption with miR-29b and *DNMT3B* mRNA Expression in the Development of Lung Cancer

**DOI:** 10.3390/genes13050836

**Published:** 2022-05-07

**Authors:** Chia-Chen Huang, Chung-Yu Lai, I-Hsin Lin, Chin-Hung Tsai, Shi-Mei Tsai, Kit-Lai Lam, Jiun-Yao Wang, Chun-Chieh Chen, Ruey-Hong Wong

**Affiliations:** 1 Department of Public Health, Chung Shan Medical University, Taichung 40201, Taiwan; cchuang1206@gmail.com (C.-C.H.); july3e06@hotmail.com (K.-L.L.); 2 Institute of Medicine, Chung Shan Medical University, Taichung 40201, Taiwan; lai1228@seed.net.tw (C.-Y.L.); jiunyao.wang@msa.hinet.net (J.-Y.W.); 3 Thoracic Surgery, Cheng Ching General Hospital, Taichung 40764, Taiwan; 4 Department of Epidemiology and Biostatistics, Memorial Sloan-Kettering Cancer Center, New York, NY 10065, USA; alleycat19850820@gmail.com; 5 Division of Pulmonary and Critical Care Medicine, Department of Internal Medicine, Tungs’ Taichung MetroHarbor Hospital, Taichung 43503, Taiwan; mict6009@gmail.com; 6 Department of Biological Science and Technology, National Chiao Tung University, Hsinchu 30010, Taiwan; 7 Department of Nursing, Chung Shan Medical University Hospital, Taichung 40201, Taiwan; csha714@gmail.com; 8 Department of Family Medicine, Cheng Ching General Hospital, Taichung 40201, Taiwan; 9 Department of Family and Community Medicine, Chung Shan Medical University Hospital, Taichung 40201, Taiwan; sp954455@yahoo.com.tw; 10 Department of Occupational Medicine, Chung Shan Medical University Hospital, Taichung 40201, Taiwan; 11 School of Medicine, Chung Shan Medical University, Taichung 40201, Taiwan

**Keywords:** *DNMT3B*, miR-29b, smoking, green tea, lung cancer

## Abstract

In tumor development, increased expression of DNA methyltransferase (DNMT) has been observed. In particular, cigarette smoke and tea polyphenols may influence *DNMT3B* mRNA expression by regulating microRNA (miR)-29b expression. Herein, we designed a case–control study to evaluate the joint effects of smoking and green tea consumption, with miR-29b and *DNMT3B* mRNA expression, in lung cancer development. A total of 132 lung cancer patients and 132 healthy controls were recruited to measure miR-29b and *DNMT3B* mRNA expression in whole blood. Results revealed that lung cancer patients had lower miR-29b expression (57.2 vs. 81.6; *p* = 0.02) and higher *DNMT3B* mRNA expression (37.2 vs. 25.8; *p* < 0.001) than healthy controls. Compared to non-smokers with both higher miR-29b and lower *DNMT3B* mRNA expression, smokers with both low miR-29b and higher *DNMT3B* mRNA expression had an elevated risk of lung cancer development (OR 5.12, 95% CI 2.64–9.91). Interactions of smoking with miR-29b or *DNMT3B* mRNA expression in lung cancer were significant. Interaction of green tea consumption with miR-29b expression and *DNMT3B* mRNA expression in lung cancer was also significant. Our study suggests that smokers and green tea nondrinkers with lower miR-29b expression and higher *DNMT3B* mRNA expression are more susceptible to lung cancer development.

## 1. Introduction

Lung cancer is the leading cause of cancer-related death worldwide [1], and people with a smoking history are most likely to develop this cancer [2]. Researches demonstrate that cigarette smoke could cause an increase in oxidative stress in lung tissues, leading to lung cancer [3,4]. On the other hand, green tea has attracted a great deal of attention as a natural product possessing preventive effects of strong antioxidants and medicinal properties [5], and it is reputed to have the potential to inhibit the development of various cancers [6,7,8]. Our previous study reported that individuals who never drink green tea have an elevated lung cancer risk compared to those who drink at least one cup of green tea per day [9]. Moreover, the effect mentioned above is more pronounced in smokers than in non-smokers [9].

Epigenetics, such as DNA methylation, refers to changes in gene expression or cellular phenotype without altering the underlying DNA sequence [10]. Typically, methylation modification occurs in the gene promoter region, especially in the CpG dinucleotide [11]. Evidence has indicated that DNA methylation can inhibit gene expression by directly or indirectly influencing the binding of the protein or the transcription factor to the promoter region [11]. Research has also highlighted that hypermethylated DNA can phenocopy loss of function mutations in tumor suppressor genes (TSG) by silencing gene expression and regulating the cell cycle genes [12,13], thereby causing cancer in some sites [14,15]. Specifically, it has been pointed out that cigarette smoking arouses DNA methylation [16,17], causing the occurrence of several cancers, including lung cancer [16,17]. Cigarette smoke can also induce the accumulation of high levels of DNA methyltransferase (DNMT) in the nucleus [18]. Such an observed phenomenon might be associated with epigenetic silencing of TSG and lead to carcinogenesis [19]. In the process of DNA methylation, DNMT is a crucial catalyst [11,12,13,14]. The DNMT isoforms (DNMT3A and DNMT3B) have similar affinities for unmethylated and hemimethylated DNA substrates, which function as the de novo DNMT to affect the methylation status of normally unmethylated CpG sites [20]. Further, our previous study observed that the *DNMT3B*-149 TT genotype with higher promoter activity could increase the lung cancer risk elicited by smoking [21]. However, researchers have not yet elucidated the effect of *DNMT3B* mRNA expression in the development of lung cancer.

MicroRNAs (miR) are an abundant class of small RNAs that play prominent roles in gene regulation [22]. Notably, miR-29b acts as both a tumor suppressor factor and regulator by influencing apoptosis, methylation, invasion and proliferation in cancers [23]. Compared to that in normal lung tissue, miR-29b in lung cancer tissue has a lower expression level [24]. More notably, miR-29b could directly target the 3′ untranslated region (UTR) of *DNMT3B*, resulting in the demethylation of TSG and influencing cell apoptosis [25,26]. However, studies have not explored the effect of simultaneous miR-29b and *DNMT3B* mRNA expression in the development of lung cancer.

Due to the presence of natural phytochemicals, including catechins/epicatechins, green tea is also known as green tea polyphenols [27]. Green tea polyphenols could inhibit *DNMT3B* mRNA expression and thus reduce tumors in different tissues or cancer cells [28,29]. A recent experimental study observed that green tea polyphenols rescued UVB-induced miR-29 depletion and prevented tumor growth by maintaining reduced DNA hypermethylation and activating tumor suppressors [30]. An in vivo study also revealed that green tea polyphenols could decrease nicotine-induced cell proliferation, β-catenin nuclear expression, and phospho-Akt levels in non-small cell lung cancer cells [31]. Further, the lower expression of the complex of T-cell factor (TCF4) and β-catenin can downregulate the expression of *c-Myc* in lung cancer [32], while forced expression of *c-Myc* can repress miR-29b [33]. However, the effect of green tea intake on the simultaneous expression of miR-29b and *DNMT3B* mRNA, which may be engaged in the development of lung cancer, is still unclear.

The current case–control study compared the differences in whole blood miR-29b and *DNMT3B* mRNA expression between lung cancer patients and healthy controls. In addition, this study evaluated the combined effects of cigarette smoking and green tea consumption with miR-29b and *DNMT3B* mRNA expression in the development of lung cancer.

## 2. Materials and Methods

### 2.1. Study Subjects

Our entire study conformed to the Declaration of Helsinki. The study design and final report were prepared with the approval of the institutional review boards of participating institutions (Taichung Cheng Ching Hospital: HP150043, Taichung Tungs’ Taichung MetroHarbor Hospital: 104072). All study subjects signed their informed consent before inclusion in the study.

Incident lung cancer patients (ICD-9 code 162, ICD-10 codes C33–C34) were recruited from Cheng Ching General Hospital and Tungs’ Taichung MetroHarbor Hospital in central Taiwan. Eligible cases were 20 years of age or older (range: 29–93 years). They underwent a series of examinations of pathologic stages by board-certified pathologists. According to the World Health Organization classification [34], tumor types and stages were determined. The 132 lung cancer patients included 88 (66.6%) with adenocarcinoma, 31 (23.5%) with squamous cell carcinoma, and 13 with other cancers (including small cell carcinoma and unspecific malignant cell). None of the included patients had been exposed to radiotherapy or chemotherapy. During the same period of case recruitment, controls were selected randomly among participants with no history of cancer or pulmonary diseases at the time of diagnosis, including tuberculosis, pneumonia, bronchiectasis, pneumoconiosis, pulmonary alveolar pneumopathy, chronic obstructive pulmonary disease, and asthma. They were admitted to the same hospitals for physical check-ups. One control was selected to individually match with each cancer patient by age (initially ± one year, relaxed to ± five years) and gender. They came from the same geographic areas as the lung cancer cases. There were no familial relationships among and between cases and controls.

### 2.2. Epidemiologic Information

Epidemiologic information was collected from in-person interviews using a standardized questionnaire as presented in our previous work [9]. The cumulative smoking dose indicator of “pack-years” was calculated as the number of cigarette packs smoked daily multiplied by the number of active smoking years. The evaluation of green tea consumption was based on a previous study [35], and we defined a standardized cup of tea as 100–120 mL. The exposure period was assessed from birth to the day when lung cancer was first diagnosed for cases or when the interview was conducted for controls. To prevent misclassification of tea intake for individuals who changed their tea consumption after diagnosis, we collected only cumulative amounts before disease onset. Subjects who reported drinking green tea were further asked to identify the length (number of years) and frequency of green tea consumption as every day (≥1 cup per day), 3–4 cups per week, 1–2 cups per week, 1–2 cups per month, and seldom. Those who consumed tea every day were also asked about the number of cups they drank: 1–2, 3–4, 5–9, or 10+ per day. Fruit and vegetable intake was measured as the average number of standardized servings per week of fruits and vegetables over the last 3 years. For cooking exposures, subjects were asked about the frequency of using various cooking methods, such as stir-frying. Family history of lung cancer referred to lung cancer in first-degree relatives of the test subject.

### 2.3. Laboratory Analyses

Total RNA, including small RNA, was extracted from whole blood using a PAXgene Blood RNA kit (Qiagen, Hilden, Germany) according to the manufacturer’s protocol and treated with DNase (Qiagen). At 260 and 280 nm, the absorbances of RNA were quantified spectrophotometrically with the NanoDrop ND-1000 (NanoDrop, Wilmington, DE, USA). Sound and good integrity and purity of RNA were defined as having an A260/A280 ratio from 1.8 to 2.0. Samples were stored at −80 °C.

Reverse transcription (RT) and quantitative PCR (qPCR) kits made specifically for accurate miRNA analysis (Applied Biosystems, Waltham, MA, USA) were used to evaluate expression of miR-29b. The 15 µL RT reactions were performed using a TaqMan microRNA Reverse Transcription Kit (Applied Biosystems) and incubated for 30 min at 16 °C, 30 min at 42 °C, and 5 min at 85 °C, and then maintained at 4 °C. For real-time PCR, 1 µL diluted RT products were mixed with 5 µL of 2× TaqMan PCR master mixture (No AmpErase UNG, Applied Biosystems), 0.4 µL TaqMan MicroRNA Assay (Applied Biosystems) and 3.6 µL nuclease-free water in a final volume of 10 µL. All reactions were run on the Step One Plus Real Time PCR System (Applied Biosystems) using the following conditions: 95 °C for 10 min, followed by 40 cycles at 95 °C for 15 s, and 60 °C for 1 min. Real-time PCR was performed in triplicate, including no-template controls. Relative expression of miR-29b was calculated using the comparative cycle threshold (CT; 2^−ΔΔCT^) method [36] with *RNU6B* as the endogenous control to normalize the data.

The expression of *DNMT3B* mRNA was measured using KAPA SYBR FAST One-Step quantitative real-time PCR Kit (Kapa Biosystems, Wilmington, MA, USA). Reactions were performed in 15 µL containing 1.87 µL mRNA, 7.5 µL 2× KAPA SYBR FAST qPCR Master Mix, 0.75 µL forward primer, 0.75 µL reverse primer, 0.3 µL 10 mM dUTP, 0.3 µL 50× KAPA RT mix, and 3.5 µL RNase-free H_2_O. The protocol used was 5 min at 42 °C and 3 min at 95 °C, followed by 40 cycles of 3 s at 95 °C and 30 s at 60 °C. *GAPDH* was used as an internal control. Target sequences were amplified with different sets of primers: *DNMT3B* primer, forward 5′-TAT CCG CAC CCC GGA GAT-3′, reverse 5′-ATC GCC TGT CAA GTC CTG TGT-3′; *GAPDH*, forward 5′-GGA GCC AAA AGG GTC ATC ATC-3′, reverse 5′-GAT GGC ATG GAC TGT GGT CAT-3′. The relative amounts of the target gene, standardized against the amount of *GAPDH*, were expressed as ΔCt = Ct(target) − Ct(*GAPDH*). The ratio of *DNMT3B* mRNA copies to *GAPDH* copies was then calculated as 2^−ΔΔCT^ [36].

### 2.4. Statistical Analysis

Comparisons were made using Student’s *t*-test for continuous variables and χ^2^-test or Fisher’s exact test for discrete variables. The expression levels of miR-29b (*p* < 0.001) and *DNMT3B* mRNA (*p* < 0.001), which were evaluated by the Kolmogorov–Smirnov test, did not confirm normal distribution. Therefore, miR-29b and *DNMT3B* mRNA expression levels were presented by median (minimum–maximum) among the basic characteristics, and compared using the Mann–Whitney *U* test or Kruskal–Wallis test. The medians of miR-29b and *DNMT3B* mRNA expression among healthy controls were used for assorting higher expression and lower expression, and examined with a log-linear regression model to obtain the odds ratio (OR) and 95% confidence interval (CI). In addition, likelihood ratio χ^2^-tests were utilized to test the interaction of smoking, green tea consumption, and miR-29b and *DNMT3B* mRNA expression levels concerning the risk of lung cancer. Interaction was further assessed using the likelihood ratio test to calculate χ^2^ and *p* values. All *p* values were calculated using two-tailed statistical tests, and a value < 0.05 was considered statistically significant. SAS 9.4 for Windows (SAS Inc., Cary, NC, USA) was used for the analyses.

## 3. Results

Basic characteristics of lung cancer and healthy controls are given in Table 1. Males accounted for 62.9% of all subjects. The average ages of lung cancer patients and healthy controls were 63.9 ± 11.6 (SD) years and 62.1 ± 11.9 years, respectively (*p* = 0.19, *t*-test). The patients had a more significant proportion of smokers (55.3%) than the controls (32.6%); 32.6% of the patients had smoked more than 40 pack-years, but only 12.9% of controls had smoked more than 40 pack-years (OR 2.51, 95% CI 1.70–3.70). More green tea nondrinkers were present among the lung cancer patients than among the healthy controls (68.1% vs. 55.3%, OR 1.67, 95% CI 1.19–2.36). Moreover, compared to healthy controls, lung cancer patients had a higher frequency of family history of lung cancer (OR 1.71, 95% CI 1.05–2.79). No significant differences were found in vegetable and fruit intake and exposure to cooking fumes when comparing the lung cancer cases and the controls.

Expression levels of miR-29b and *DNMT3B* mRNA in the groups of lung cancer patients and healthy controls are shown in Table 2. Lung cancer patients had significantly lower miR-29b expression than healthy controls (57.2 vs. 81.6, *p* = 0.02, Mann–Whitney *U* test). In the case group, significant differences in miR-29b expression were observed in the different strata of gender (*p* < 0.001), age (*p* = 0.04), smoking status (*p* < 0.001), pack-years smoked (*p* < 0.001), and family history of lung cancer (*p* = 0.02). Contrarily, lung cancer patients had significantly higher *DNMT3B* mRNA expression than healthy controls (37.2 vs. 25.8, *p* < 0.001). In the case group, significant differences in *DNMT3B* mRNA expression were also observed in the different strata of gender (*p* < 0.001), age (*p* = 0.002), smoking status (*p* < 0.001), pack-years smoked (*p* < 0.001), and green tea consumption (*p* < 0.001). However, the respective relationships between nearly all analyzed variables and miR-29b expression and *DNMT3B* mRNA expression in lung cancer patients showed a different direction compared with the results in healthy controls. In addition, cancer-free smokers had significantly lower miR-29b expression (*p* < 0.001) and non-significant but slightly higher *DNMT3B* mRNA expression (*p* = 0.45) when compared to cancer-free nonsmokers.

We evaluated the joint effects of miR-29b and *DNMT3B* mRNA expression in the development of lung cancer (Table 3). The medians of miR-29b and *DNMT3B* mRNA expression among healthy controls were used to assort higher or lower expression. Furthermore, subjects with both higher miR-29b expression and lower *DNMT3B* mRNA expression were selected as the reference group. After adjusting the effects of pack-years smoked, green tea consumption, exposure to fumes of cooking, and family history of lung cancer, those subjects with both lower miR-29b expression and higher *DNMT3B* mRNA expression, those with both higher miR-29b expression and higher *DNMT3B* mRNA expression, and those with both lower miR-29b expression and lower *DNMT3B* mRNA expression had a 2.59-fold (95% CI 1.56–4.31), 1.62-fold (95% CI 0.97–2.72), and 1.75-fold (95% CI 1.06–2.90) increased risk of lung cancer compared to the reference group, respectively. Moreover, subjects with lower miR-29b expression and higher *DNMT3B* mRNA expression, those with higher miR-29b expression and higher *DNMT3B* mRNA expression, and those with lower miR-29b expression and lower *DNMT3B* mRNA expression were combined to increase the statistical power; these subjects also had significant risk of lung cancer (OR 1.95, 95% CI 1.23–3.10). However, no significant interaction between miR-29b and *DNMT3B* mRNA expression levels in the development of lung cancer was observed.

Subsequently, we evaluated the joint effects of smoking status with miR-29b and *DNMT3B* mRNA expression in the development of lung cancer (Table 4). After adjusting the confounding effect, smokers with lower miR-29b expression (OR 3.95, 95% CI 2.37–6.59), smokers with higher miR-29b expression (OR 6.09, 95% CI 3.35–11.07), and never-smokers with lower miR-29b expression (OR 3.27, 95% CI 2.06–5.19) had an increased risk of lung cancer compared to never-smokers with higher miR-29b expression. Interestingly, a significant interaction between smoking status and miR-29b expression was observed in lung cancer development (*p* < 0.001). When miR-29b was replaced by *DNMT3B*, smokers with higher *DNMT3B* mRNA expression (OR 2.96, 95% CI 1.94–4.50) and smokers with lower *DNMT3B* mRNA expression (OR 1.17, 95% CI 0.72–1.89) also had an increased risk of lung cancer compared to non-smokers with lower *DNMT3B* mRNA expression. A significant interaction between smoking status and *DNMT3B* mRNA expression in lung cancer development was also observed (*p* = 0.001). Furthermore, non-smokers with both higher miR-29b and lower *DNMT3B* mRNA expression levels were regarded as the reference. Smokers with both lower miR-29b and higher *DNMT3B* mRNA expression levels (OR 5.12, 95% CI 2.64–9.91), and smokers with both higher miR-29b and higher *DNMT3B* mRNA expression levels (OR 6.88, 95% CI 3.27–14.46) had an evident risk of lung cancer. Even among non-smokers, those with lower miR-29b expression and higher *DNMT3B* mRNA expression also had a higher risk of developing lung cancer (OR 3.18, 95% CI 1.65–6.15). A significant interaction between smoking status and miR-29b and *DNMT3B* mRNA expression levels in lung cancer development remained (*p* < 0.001).

The combined effects of green tea consumption with miR-29b and *DNMT3B* mRNA expression on lung cancer development were also evaluated (Table 5). After the confounding effects were adjusted, green tea nondrinkers with lower miR-29b expression had a 1.75-fold (95% CI 1.15–2.67) increased risk of lung cancer compared to green tea drinkers with higher miR-29b expression. Similarly, green tea nondrinkers with higher *DNMT3B* mRNA expression had a 1.42-fold (95% CI 0.94–2.14) higher risk of lung cancer than green tea drinkers with lower *DNMT3B* mRNA expression. The interaction between green tea consumption and *DNMT3B* mRNA expression in lung cancer development was significant (*p* < 0.001). Furthermore, green tea drinkers with both higher miR-29b and lower *DNMT3B* mRNA expression levels were regarded as the reference. Green tea nondrinkers with both lower miR-29b and higher *DNMT3B* mRNA expression levels had a 3.23-fold (95% CI 1.55–6.72) increased risk of lung cancer, and this interaction of green tea consumption with miR-29b and *DNMT3B* mRNA expression was significant in lung cancer development (*p* < 0.001).

## 4. Discussion

Our study found that subjects with lower miR-29b expression or higher *DNMT3B* mRNA expression had a significantly increased risk of lung cancer. The interactions of cigarette smoking with miR-29b and *DNMT3B* mRNA expression levels were both significant in the development of lung cancer. The joint effect of green tea consumption with miR-29b and *DNMT3B* mRNA expression was also evident in the development of lung cancer.

It has been observed that miR-29b can directly target *DNMT3B*, and miR-29b expression is inversely correlated to *DNMT3B* expression in lung cancer tissues [25]. Furthermore, the increased miR-29b expression can also restore common DNA methylation patterns in lung cancer cell lines, inducing re-expression of methylation-silenced TSGs to suppress the tumor development [25]. However, the effect of simultaneous miR-29b and *DNMT3B* mRNA expression in the development of lung cancer has not been explored. In the present study, lung cancer patients had significantly lower miR-29b expression and significantly higher *DNMT3B* mRNA expression than did healthy controls. Further, subjects with lower miR-29b expression or *DNMT3B* mRNA expression had significantly increased risk of lung cancer compared to subjects with both higher miR-29b expression and lower *DNMT3B* mRNA expression; however, no significant interaction between miR-29b and *DNMT3B* mRNA was observed in the development of lung cancer. Such results reflect that individuals with low miR-29b expression or high *DNMT3B* mRNA expression alone are likely to have increased risk of developing lung cancer. However, the sample size in the current study was too small to detect the interaction effect of miR-29b and *DNMT3B* mRNA expressions in lung cancer development.

In the current study, miR-29b and *DNMT3B* mRNA expression levels were related to smoking status. We further observed that cancer-free smokers had significantly lower miR-29b expression and non-significant but slightly higher *DNMT3B* mRNA expression than cancer-free non-smokers did. Previously, an experimental study showed that β-catenin expression is increased in the airway epithelium in chronic obstructive pulmonary disease, mainly due to cigarette smoking [37]. Additionally, the complex of TCF4 and β-catenin can directly induce *c-Myc* protein to inhibit miR-29b expression [33]. Further, NNK was demonstrated to reduce the degradation of the β-transducin repeat-containing protein (βTrCP) by the AKT pathway and induce βTrCP translocation to the cytoplasm, resulting in DNMT1 nuclear accumulation and hypermethylation of the promoters of TSGs [18]. Crucially, AKT kinase could be activated by smoking compounds through an ROS-dependent mechanism [38]. Even if our results can be supported by biological evidence, more research is needed to confirm them, especially to clarify the role of ROS-related signaling pathways. It should also be noted that smokers with lung cancer showed significantly higher miR-29b expression than nonsmokers with lung cancer in this study. It must be considered that subjects may also have been exposed to other substances that attenuate miR-29b expression that was not identified in this study. Therefore, the observed miR-29b expression should be regarded as the overall effect of explored and unexplored factors. However, it is questionable whether a single measurement can adequately represent the participants’ exposure to carcinogens. Another explanation for the current observations could be the possibility of recall bias in self-reported smoking data, thereby causing exposure misclassification.

Currently, our study highlights the significant interaction of smoking with miR-29b expression, and that of smoking with *DNMT3B* mRNA expression in lung cancer development. Such epidemiological observations can be reasonably explained by biological evidence. Lower miR-29b expression or higher *DNMT3B* mRNA expression might cause higher DNA methylation [23,25]. In particular, smokers are not only exposed to the carcinogens (such as NNK), which influence *DNMT3B* mRNA expression [18], but are also exposed to other carcinogens that cause associated effects [3,18]. Therefore, smokers with lower miR-29b expression or higher *DNMT3B* mRNA expression are more likely to develop lung cancer.

Furthermore, we observed that smokers with lower miR-29b and higher *DNMT3B* mRNA expression levels had a more pronounced lung cancer risk than other combinations of smoking status with miR-29b and *DNMT3B* mRNA expression, except for smokers with higher miR-29b and higher *DNMT3B* mRNA expression levels. Even though non-smokers are not exposed to cigarettes, those with lower miR-29b expression and higher *DNMT3B* mRNA expression risk developing lung cancer. It seems that subjects who have lower miR-29b and/or higher *DNMT3B* mRNA expression are likely to reveal lung cancer risk regardless of cigarette exposure. When effects of smoking status, miR-29b, and *DNMT3B* mRNA expression were further assessed simultaneously, we also found significant interaction between these three factors in lung cancer development. These results indicate that each influencing factor produces a different risk of lung cancer development; when they are combined together, a more prominent risk may present. Because of the relatively few subjects in the statistical stratification of this study, there may be insufficient statistical power. Additional studies including more subjects may shed light on this question.

In the present study, green tea consumption was observed to be associated with *DNMT3B* mRNA expression, but not miR-29b expression, especially in lung cancer patients with higher *DNMT3B* mRNA expression. The green tea consumption-associated potential effects have been shown to contribute to the antioxidant capacity of tea polyphenols [27]. In particular, epigallocatechin-3-gallate (EGCG), the main constituent of green tea, can inhibit DNMT activity and reactivate methylation-silenced genes [28,29]. Previously, a study showed that tea polyphenols significantly reduce hypermethylation in lung cancer [39]. A recent study has also pointed out that green tea can affect the expression of miR-29b [30]. However, through our current epidemiological observations, the effect of green tea on the expression of miR-29b may be indirect and vague [31]. In contrast, its impact on the expression of *DNMT3B* may be more direct and more apparent. Our observations show evidence that tea polyphenols emerge as putative preventives and coadjuvants in the treatment of lung cancer related to DNA methylation. However, such speculation needs to be confirmed. Notably, a significant interaction between green tea consumption and *DNMT3B* mRNA expression was also observed in lung cancer development. Such results suggest that subjects who do not drink green tea have lower scavenging ability for free radicals and carcinogens. Once they have a higher level of DNA methylation, they are more likely to develop lung cancer. Extending our thinking, subjects with the above conditions, coupled with lower miR-29b expression, were more likely to develop lung cancer, echoing our findings that three factors (green tea consumption, *DNMT3B* mRNA expression and miR-29b expression) have significant interaction in the development of lung cancer. However, future studies should examine the mechanisms of our results in lung cancer development.

In particular, the relationship between the analyzed variables, miR-29b expression, and *DNMT3B* mRNA expression, showed different directions in the respective groups of lung cancer cases and healthy controls. The exact reason is unknown. Future studies require further investigation of whether miR-29b and *DNMT3B* mRNA expression are inversely affected by lung cancer progression. In addition, age and gender were associated with miR-29b and *DNMT3B* mRNA expression in our lung cancer subjects. Unfortunately, there are no data in the medical literature regarding actual age and gender differences.

In the current study, the expression levels of miR-29b and *DNMT3B* mRNA were measured by real-time quantitative PCR, and the differences in the Ct values in triplicate had to be less than 0.5. Therefore, our RNA expression data had good reliability. In the current study, the exposure data of study subjects were obtained through questionnaires. Recall bias may be a problem. Thus, misclassification of exposure may have influenced the effect of specific variables on miR-29b expression, *DNMT3B* mRNA expression, and lung cancer risk. In addition, statistical analyses were further stratified, and smaller sample sizes may have limited the statistical power to detect associations between specific variables and lung cancer risk. More extensive studies should further elucidate our results.

Our results suggested that smokers and green tea nondrinkers with lower miR-29b expression and higher *DNMT3B* mRNA expression are more susceptible to lung cancer development.

## Figures and Tables

**Table 1:** Basic characteristics of lung cancer patients and controls.

	Cases	Controls	
Variables	*n* = 132	*n* = 132	OR (95% CI) ^b^
Gender			
Male	83 (62.9%)	83 (62.9%)	0.98 (0.76–1.26)
Female	49 (37.1%)	49 (37.1%)	1.00
Age at recruitment (years, mean ± SD)	63.9 ± 11.6	62.1 ± 11.9	
≥60	86 (65.2%)	79 (59.9%)	1.16 (0.62–2.17)
51–59	30 (22.7%)	28 (21.2%)	1.24 (0.78–1.96)
≤50	16 (12.1%)	25 (18.9%)	1.00
Smoking status			
Current and ever smokers	73 (55.3%)	43 (32.6%)	2.07 (1.49–2.86) ***
Non-smokers	59 (44.7%)	89 (67.4%)	1.00
Pack-years smoked			
≥40	43 (32.6%)	17 (12.9%)	2.51 (1.70–3.70) ***
1–39	30 (22.7%)	26 (19.7%)	1.72 (1.19–2.50) **
0	59 (44.7%)	89 (67.4%)	1.00
Green tea consumption (cups/day)			
0	90 (68.1%)	73 (55.3%)	1.67 (1.19–2.36) **
<1	27 (20.5%)	23 (17.4%)	1.69 (1.12–2.56) *
≥1	15 (11.4%)	36 (27.3%)	1.00
Green tea consumption (years)			
0	90 (68.1%)	73 (55.3%)	1.27 (0.92–1.75)
≤10	20 (15.2%)	29 (22.0%)	0.98 (0.66–1.46)
>10	22 (16.7%)	30 (22.7%)	1.00
Fruit and vegetable intake (servings/week)			
≤14	38 (28.8%)	51 (38.6%)	0.84 (0.63–1.11)
15–20	32 (24.2%)	23 (17.4%)	1.16 (0.84–1.60)
≥21	62 (47.0%)	58 (44.0%)	1.00
Exposure to cooking fumes (hours/week)			
≥3	13 (9.9%)	5 (3.8%)	1.71 (0.99–2.94) ^c^
1–3	8 (6.1%)	8 (6.1%)	1.09 (0.64–1.84)
<1	111 (84.0%)	119 (90.1%)	1.00
Family history of lung cancer			
Yes	16 (12.1%)	6 (4.6%)	1.71 (1.05–2.79) *
No	116 (87.9%)	126 (95.4%)	1.00
Histological type			
Adenocarcinoma	88 (66.6%)		
Squamous cell carcinoma	31 (23.5%)		
Others ^a^	13 (9.9%)		

OR, odds ratio; CI, confidence interval. ^a^ Included small cell lung cancer (*n* = 3), adenosquamous carcinoma (*n* = 2) and unclassified carcinoma (*n* = 8). ^b^ Data were matched by age and gender, and calculated by log-linear regression. ^c^ *p*-Value = 0.056. * 0.01 < *p* < 0.05, ** 0.001 < *p* < 0.01, *** *p* < 0.001.

**Table 2:** Expression levels of miR-29b and *DNMT3B* mRNA in the groups of lung cancer patients and controls stratified by basic characteristics.

	miR-29b Expression					DNMT3B mRNA				
	Cases		Controls			Cases		Controls		
Variables	*n*	Median (Min–Max)	*n*	Median (Min–Max)	*p*-Value ^b^	*n*	Median (Min–Max)	*n*	Median (Min–Max)	*p*-Value ^b^
Total	132	57.2 (0.4–1317.6)	132	81.6 (0.01–15,100.2)	0.02	132	37.2 (0.4–920.0)	132	25.8 (0.01–1972.8)	<0.001
Gender										
Male	83	68.5 (0.6–1317.6)	83	75.2 (0.01–6400.0)	0.88	83	63.1 (0.4–920.0)	83	23.6 (0.01–1972.8)	<0.001
Female	49	40.1 (0.4–409.9)	49	90.6 (5.2–15,100.2)	<0.001	49	24.5 (1.9–146.3)	49	29.8 (1.3–440.3)	0.09
*p*-Value ^c^		<0.001		0.24			<0.001		0.07	
Age at recruitment (years)										
≥60	86	63.5 (0.6–1317.6)	79	75.8 (6.1–15,100.2)	0.27	86	56.0 (0.4–544.0)	79	21.2 (0.01–1972.8)	<0.001
51–59	30	43.4 (0.9–1032.3)	28	48.9 (0.01–768.6)	0.78	30	24.8 (2.6–920.0)	28	30.8 (1.3–165.7)	0.97
<50	16	34.9 (0.4–241.3)	25	160.4 (5.2–6400.0)	<0.001	16	24.1 (4.8–60.9)	25	35.6 (3.4–440.3)	0.02
*p*-Value ^c^		0.04		<0.001			0.002		<0.001	
Smoking status										
Current and ever smokers	73	74.1 (3.7–1317.6)	43	31.3 (0.01–6400.0)	<0.001	73	66.5 (2.6–920.0)	43	27.0 (12.2–190.1)	<0.001
Non-smokers	59	38.7 (0.4–409.9)	89	96.5 (6.1–15,100.2)	<0.001	59	24.5 (0.4–146.3)	89	25.5 (0.01–1972.8)	0.84
*p*-Value ^c^		<0.001		<0.001			<0.001		0.45	
Pack-years smoked										
≥40	43	75.7 (3.7–1317.6)	17	27.1 (9.5–182.2)	<0.001	43	67.8 (2.6–196.6)	17	24.3 (12.3–144.9)	0.001
1–39	30	68.5 (3.8–558.1)	26	37.8 (0.01–6400.0)	0.04	30	61.7 (10.0–920.0)	26	29.2 (12.2–190.1)	0.004
0	59	38.7 (0.4–409.9)	89	96.5 (6.1–15,100.2)	<0.001	59	24.5 (0.4–146.3)	89	25.5 (0.01–1972.8)	0.84
*p*-Value ^c^		<0.001		<0.001			<0.001		0.62	
Green tea consumption (cups/day)										
0	90	56.4 (0.4–1032.3)	73	89.3 (9.5–15,100.2)	0.003	90	54.5 (2.6–920.0)	73	21.2 (1.3–204.8)	<0.001
<1	27	63.8 (0.9–402.3)	23	72.3 (5.2–6400.0)	0.91	27	25.1 (0.4–205.1)	23	35.6 (3.4–1972.8)	0.29
≥1	15	49.8 (3.7–1317.6)	36	76.1 (0.01–794.3)	0.88	15	23.6 (11.9–196.6)	36	35.5 (0.01–440.3)	0.18
*p*-Value ^c^		0.91		0.34			<0.001		<0.001	
Green tea consumption (years)										
0	90	56.4 (0.4–1032.3)	73	89.3 (9.5–15,100.2)	0.003	90	54.5 (2.6–920.0)	73	21.2 (1.3–204.8)	<0.001
≤10	20	47.2 (9.7–402.3)	29	58.8 (0.01–794.3)	0.82	20	24.4 (1.9–205.1)	29	32.2 (3.4–440.3)	0.28
>10	22	65.8 (0.9–1317.6)	30	84.2 (5.2–6400.0)	0.49	22	25.7 (0.4–196.6)	30	35.7 (0.01–1972.8)	0.13
*p*-Value ^c^		0.83		0.15			<0.001		<0.001	
Fruit and vegetable intake (servings/week)										
≤14	38	63.3 (3.8–1032.3)	51	77.1 (12.2–15,100.2)	0.3	38	51.6 (1.9–920.0)	51	26.0 (1.3–165.0)	0.002
15–20	32	42.0 (0.4–478.2)	23	89.8 (0.01–6400.0)	0.1	32	28.9 (0.4–165.7)	23	25.5 (3.4–190.1)	0.4
≥21	62	57.7 (0.6–1317.6)	58	83.7 (6.1–2793.4)	0.24	62	38.0 (3.4–544.0)	58	25.6 (0.01–1972.8)	0.01
*p*-Value ^c^		0.27		0.95			0.17		0.99	
Exposure to cooking fumes (hours/week)										
≥3	13	42.7 (29.0–231.7)	5	142.7 (118.7–164.3)	0.05	13	29.3 (10.0–910.8)	5	32.4 (10.8–35.9)	0.77
1–3	8	43.8 (31.0–154.9)	8	124.2 (39.2–794.3)	0.004	8	24.0 (13.5–70.6)	8	23.9 (1.3–95.5)	1
<1	111	62.3 (0.4–1317.7)	119	77.1 (0.01–15,100.2)	0.14	111	43.5 (0.4–920.0)	119	25.5 (0.01–1972.8)	<0.001
*p*-Value ^c^		0.46		0.04			0.24		0.9	
Family history of lung cancer										
Yes	16	41.3 (0.6–155.6)	6	59.6 (13.3–571.4)	0.49	16	22.1 (1.9–87.7)	6	9.4 (0.01–31.6)	0.06
No	116	63.3 (0.4–1317.6)	126	81.7 (0.01–15,100.2)	0.07	116	40.0 (0.4–920.0)	126	26.7 (1.3–1972.8)	<0.001
*p*-Value ^c^		0.02		0.42			0.45		0.004	
Histological type										
Adenocarcinoma	88	56.4 (0.4–1317.6)				88	30.1 (1.9–920.0)			
Squamous cell carcinoma	31	51.8 (0.9–305.7)				31	60.9 (0.4–910.8)			
Others ^a^	13	69.0 (4.0–231.7)				13	67.7 (11.1–196.0)			
*p*-Value ^c^		0.45					0.09			

^a^ Included small cell lung cancer (*n* = 3), adenosquamous carcinoma (*n* = 2) and unclassified carcinoma (*n* = 8); ^b^ Mann–Whitney *U* test was performed to examine the differences in miR-29b and *DNMT3B* mRNA expression between the case and control groups for each variable; ^c^ Mann–Whitney *U* test and Kruskal–Wallis test were performed to examine the differences in miR-29b and *DNMT3B* mRNA expression in the case and control groups for each variable.

**Table 3:** Joint effects of miR-29b and *DNMT3B* mRNA in lung cancer development.

	Cases	Controls					
Variables	*n* = 132	*n* = 132	OR (95% CI)	*p*-Value ^a^		OR (95% CI)	*p*-Value ^a^
miR-29b/*DNMT3B* mRNA							
Low/High ^b^	58 (44.0%)	29 (22.0%)	2.59 (1.56–4.31)	<0.001	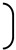		
High/High	30 (22.7%)	37 (28.0%)	1.62 (0.97–2.72)	0.07		1.95 (1.23–3.10)	0.005
Low/Low	37 (28.0%)	37 (28.0%)	1.75 (1.06–2.90)	0.03			
High/Low	7 (5.3%)	29 (22.0%)	1.00 (reference)			1.00 (reference)	
Test for interaction	χ^2^ = 0.09 (1 df); *p* = 0.76						

OR, odds ratio; CI, confidence interval. ^a^ Data were matched by age and gender, calculated by log-linear regression, adjusted for pack-years smoked, green tea consumption, exposure to fumes of cooking, and family history of lung cancer. ^b^ Cut-off points were determined according to the medians of miR-29b and *DNMT3B* mRNA among healthy controls. *RNU6B* and *GAPDH* were used as the endogenous controls for miR-29b and *DNMT3B* mRNA, respectively.

**Table 4:** Joint effects of smoking status with miR-29b and *DNMT3B* mRNA in lung cancer development.

	Cases	Controls		
Variables	*n* = 132	*n* = 132	OR (95% CI)	*p*-Value ^a^
Smoking status/miR-29b				
Smokers/Low	45 (34.1%)	33 (25.0%)	3.95 (2.37–6.59)	<0.001
Smokers/High	28 (21.2%)	10 (7.6%)	6.09 (3.35–11.07)	< 0.001
Non-smokers/Low	50 (37.9%)	33 (25.0%)	3.27 (2.06–5.19)	<0.001
Non-smokers/High	9 (6.8%)	56 (42.4%)	1.00 (reference)	
Test for interaction	χ^2^ = 27.73 (1 df); *p* < 0.001			
Smoking status/*DNMT3B* mRNA				
Smokers/High	61 (46.2%)	22 (16.7%)	2.96 (1.94–4.50)	<0.001
Smokers/Low	12 (9.1%)	21 (15.9%)	1.17 (0.72–1.89)	0.53
Non-smokers/High	27 (20.5%)	44 (33.3%)	0.94 (0.65–1.37)	0.77
Non-smokers/Low	32 (24.2%)	45 (34.1%)	1.00 (reference)	
Test for interaction	χ^2^ = 10.89 (1 df); *p* = 0.001			
Smoking status/miR-29b/*DNMT3B* mRNA				
Smokers/Low/High	36 (27.3%)	16 (12.1%)	5.12 (2.64–9.91)	<0.001
Smokers/Low/Low	9 (6.8%)	17 (12.9%)	2.04 (1.00–4.15)	0.054
Smokers/High/High	25 (18.9%)	6 (4.6%)	6.88 (3.27–14.46)	<0.001
Smokers/High/Low	3 (2.3%)	4 (3.0%)	3.38 (1.26–9.08)	0.02
Non-smokers/Low/High	22 (16.7%)	13 (9.9%)	3.18 (1.65–6.15)	0.001
Non-smokers/Low/Low	28 (21.2%)	20 (15.2%)	2.79 (1.49–5.21)	0.002
Non-smokers/High/High	5 (3.8%)	31 (23.4%)	0.83 (0.38–1.83)	0.65
Non-smokers/High/Low	4 (3.0%)	25 (18.9%)	1.00 (reference)	
Test for interaction	χ^2^ = 34.26 (3 df); *p* < 0.001			
*p* for trend	<0.001			

OR, odds ratio; CI, confidence interval. ^a^ Data were matched by age and gender, calculated by log-linear regression, adjusted for green tea consumption, exposure to cooking fumes, and family history of lung cancer.

**Table 5:** Combined effects of green tea consumption with miR-29b and *DNMT3B* mRNA in lung cancer development.

	Cases	Controls		
Variables	*n* = 132	*n* = 132	OR (95% CI)	*p*-Value ^a^
Green tea consumption/miR-29b				
Nondrinkers/Low	67 (50.8%)	33 (25.0%)	1.75 (1.15–2.67)	0.01
Nondrinkers/High	23 (17.4%)	40 (30.3%)	0.98 (0.62–1.55)	0.93
Drinkers/Low	28 (21.2%)	33 (25.0%)	1.16 (0.75–1.79)	0.51
Drinkers/High	14 (10.6%)	26 (19.7%)	1.00 (reference)	
Test for interaction	χ^2^ = 2.96 (1 df); *p* = 0.09			
Green tea consumption/*DNMT3B* mRNA				
Nondrinkers/High	69 (52.3%)	27 (20.4%)	1.42 (0.94–2.14)	0.10
Nondrinkers/Low	21 (15.9%)	46 (34.8%)	0.12 (0.40–0.95)	0.03
Drinkers/High	19 (14.4%)	39 (29.6%)	0.63 (0.41–0.99)	0.05
Drinkers/Low	23 (17.4%)	20 (15.2%)	1.00 (reference)	
Test for interaction	χ^2^ = 18.21 (1 df); *p* < 0.001			
Green tea consumption/miR-29b/*DNMT3B* mRNA				
Nondrinkers/Low/High	48 (36.4%)	7 (5.3%)	3.23 (1.55–6.72)	0.0026
Nondrinkers/Low/Low	19 (14.4%)	26 (19.7%)	1.05 (0.28–2.08)	0.89
Nondrinkers/High/High	21 (15.9%)	20 (15.2%)	1.29 (0.64–2.59)	0.48
Nondrinkers/High/Low	2 (1.5%)	20 (15.2%)	0.49 (0.19–1.27)	0.15
Drinkers/Low/High	10 (7.6%)	22 (16.6%)	0.83 (0.41–1.70)	0.62
Drinkers/Low/Low	18 (13.6%)	11 (8.3%)	1.67 (0.82–3.44)	0.17
Drinkers/High/High	9 (6.8%)	17 (12.9%)	0.99 (0.48–2.06)	0.98
Drinkers/High/Low	5 (3.8%)	9 (6.8%)	1.00 (reference)	
Test for interaction	χ^2^ = 34.59 (3 df); *p* < 0.001			
*p* for trend	<0.001			

OR, odds ratio; CI, confidence interval. ^a^ Data were matched by age and gender, calculated by log-linear regression, adjusted for pack-years smoked, exposure to cooking fume, and family history of lung cancer.

## Data Availability

The data presented in this study are available on request from the corresponding author.
